# Impacts, Potential Benefits and Eradication Feasibility of Aquatic Alien Species in an Integral Natural State Reserve

**DOI:** 10.3390/biology13010064

**Published:** 2024-01-22

**Authors:** Daniele Paganelli, Adriana Bellati, Andrea Gazzola, Francesco Bracco, Daniele Pellitteri-Rosa

**Affiliations:** 1 Department of Earth and Environmental Science, University of Pavia, Via Ferrata 1, 27100 Pavia, Italy; daniele.paganelli@unipv.it (D.P.); andrea.gazzola@unipv.it (A.G.); francesco.bracco@unipv.it (F.B.); 2 Department of Ecological and Biological Sciences, University of Tuscia, Largo dell’Università snc, 01100 Viterbo, Italy; adriana.bellati@unitus.it

**Keywords:** amphibians, biodiversity, crayfish, ecosystem services, fish, gastropods, INSEAT, NNRM, management, reptiles, wetlands

## Abstract

**Simple Summary:**

Biodiversity is facing increasing threats and loss worldwide because of anthropogenic pressures. Natural reserves act as protected reservoirs for local biodiversity, and the management of alien species in these areas represents one of the main challenges for conservation biologists and environmental managers. We applied the Invasive Species Effects Assessment Tool (INSEAT) to quantify their impact and potential benefits on ecosystem services and the results indicated that all the assessed species had more negative impacts than benefits on the ecosystem services. Moreover, for each species, we evaluated the feasibility of the main eradication techniques currently proposed in the literature using the non-native risk management scheme (NNRM), with the final aim of suggesting effective actions for the management of aquatic invasive species in this kind of environment. As globalization is leading to the ever-growing spread of alien species worldwide, we stress the urgency of implementing risk assessment and management tools, particularly in protected areas, to highlight the multiple (sometimes unexpected) consequences of alien species introduction, that in turn will help address management decisions for effective conservation actions.

**Abstract:**

Riverine wetlands are stepping-stone environments for the protection of local biodiversity, but they are particularly vulnerable to biological invasions. In order to take action against biological invasions, it is crucial to assess the impacts of alien species. However, it is also important to assess the potential benefits on ecosystem services that alien species could have. Once it has been verified that negative impacts are higher than potential benefits, it is important to propose feasible actions to contrast them. In this study, we assessed eight freshwater alien species recorded in an integral protected wetland using the Invasive Species Effects Assessment Tool (INSEAT) to quantify their negative impacts and potential benefits on ecosystem services. Moreover, for each species, we evaluated the feasibility of the main eradication techniques currently proposed in the literature using the Non-Native Risk Management scheme (NNRM), with the final aim of suggesting effective actions for their management. The INSEAT results indicated that all the assessed species had more impacts than benefits while NNRM provided useful indications on the best practical conservation actions to use for reducing the density, and therefore, the negative impacts on ecosystem services and the local biodiversity of the assessed alien species.

## 1. Introduction

The EU Biodiversity Strategy for 2030 aims to halt and reverse the loss of biodiversity by defining four different pillars for effective conservation strategies: (i) expanding protected areas, (ii) restoring nature and ensuring its sustainable management across all sectors and ecosystems, (iii) strengthening the EU’s biodiversity governance framework, knowledge, research, financing and investments and (iv) deploying EU external actions to raise the level of ambition for biodiversity worldwide and reduce the impact of trade and support biodiversity outside Europe [1]. Native species are subjected to various anthropogenic pressures, such as habitat reduction, overexploitation, pollution, the spread of pathogens and biological invasions, against whom protected areas act as refuges [2,3,4].

To protect native biodiversity, it is mandatory to expand the network of protected areas, implementing ecological connections among them and favouring the movement of native species across the landscape. At the same time, it is crucial to manage protected areas effectively to prevent the introduction and subsequent spread of alien species.

Biological invasions are particularly challenging for researchers and conservationists, as they can be very hard to manage properly and timely in order to contrast the loss of biodiversity [5,6]. In addition, it has been shown that biological invasions cause severe economic problems by disrupting ecosystem services [7]. Due to the relevance of the economic impacts caused by alien species, a global database on the costs of biological invasion has been created (InvaCost database), which includes the costs associated with invasive species at different scales [8,9,10]. For example, the economic impacts of alien species in Italy in the last 30 years was estimated to be EUR 704.78 million (US 819.76 million) [11]. Although the amount of money seems very high, most of the costs are underestimated because the information is often based on grey literature, and thus is more difficult to find and be verified [11].

The lack of information is also due to the difficulty of quantifying some impacts, such as the loss of ecosystem services [12]. In this scenario, the Italian Strategy for Biodiversity [13] has proposed specific actions for managing non-native species, focusing on the control of the vectors and pathways of introduction and monitoring programs to detect the arrivals of new species early. In this framework, the implementation of eradication projects is thought to be highly advisable once alien species are detected, but only when their effectiveness is assured by reliable evaluation procedures [14].

Indeed, eradication can be very demanding in terms of money, time, energy and possible negative/unknown drawbacks for local biodiversity. Much more important, it may not always be possible from an ecological point of view. In fact, it could be feasible in relatively small and closed environments, where the alien species is detected early after its introduction and where it is not already widespread [15]. Moreover, the effectiveness of the strategy is strongly linked to the life history traits of the alien species, especially its reproduction rate which could make (if high) the eradication effort ineffective. Apart from species’ ecology, there are several other aspects to consider, such as the associated costs of the eradication strategy and, finally, public support [16,17]. Additionally, stakeholders’ support for the eradication process could be obtained only if the non-native species is not perceived as charismatic or iconic [18,19,20], if it doesn’t provide any positive impacts on the economy, and, finally, only if the eradication process does not have any collateral impacts on local fauna or natural ecosystems [21]. 

In the last few years, risk and impact assessment tools have been developed to provide a reliable evaluation of the negative impacts caused by IAS, e.g., [22]. However, such tools did not consider the potential benefits of new species release, even though a few studies showed that sometimes IAS could be somehow born by the hosting ecosystem [23]. Similarly, they did not quantify the magnitude of ecosystem modification resulting from both the positive and negative consequences of IAS [21]. Indeed, once an alien species is established, the management options are control or, when still feasible, eradication. However, both strategies have high costs, and they can be considered effective only for a limited number of IAS. 

As a result, the socio-economic aspects and the quantification of the ecosystem services provided or altered by non-indigenous species have recently attracted more attention among researchers, conservationists, and environmental managers facing biological invasions, and are becoming a fundamental part of the evaluation process for successfully managing alien species [24,25].

In this context, the aim of this paper was to provide an informative background for the effective management of several alien freshwater species recorded in a strict natural protected area by defining: (i) their potential positive and/or negative impacts on the ecosystem services using the Invasive Species Effects Assessment Tool (INSEAT [21]) and (ii) the feasibility of eradication techniques on the same alien species using the Non-Native Risk Management scheme (NNRM, [16]) to assess the effectiveness, practicability, costs, impacts, acceptability, window of opportunity and likelihood of reinvasion in the management area.

## 2. Materials and Methods

### 2.1. Study Area

**Figure 1:**
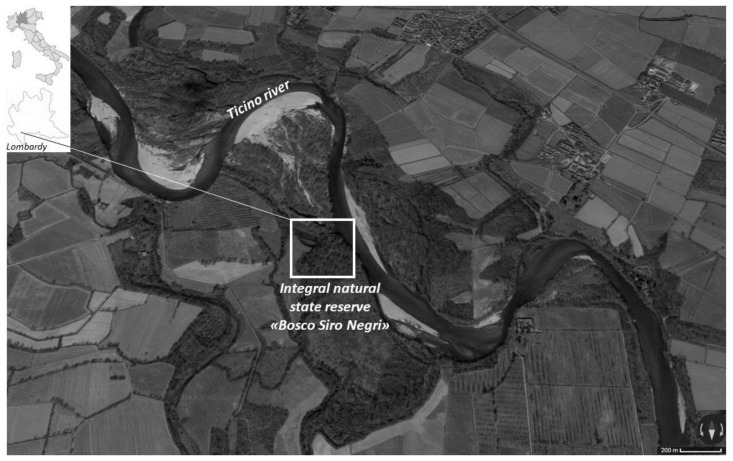
The study area: the integral natural state reserve ‘Bosco Siro Negri’ located in the province of Pavia (Lombardy, NW Italy).

The Bosco Siro Negri Strict Natural Reserve (45°21′ N; 9°05′ E, 65 m a.s.l.) is a relatively extended area (c. 34 ha) located in the southern part of the Ticino River Regional Park in northern Italy (Figure 1). The reserve is one of the few residual flood plain habitats in northern Italy, sensu Directive 92/43/EEC (Habitat 91F0 “Riparian mixed forests of *Quercus robur*, *Ulmus laevis* and *Ulmus minor*, *Fraxinus excelsior* or *Fraxinus angustifolia* (*Ulmenion minoris*) along the great rivers”). It is currently managed by the Department of Earth and Environmental Sciences of the University of Pavia, and, because of its status, no human activities are allowed in the reserve except for scientific research and surveillance. These characteristics give considerable naturalistic and scientific value to the protected area [26]. The natural reserve is located on the right bank of the Ticino River, and it is often flooded in spring and/or autumn with an estimated return period of 5–10 years, as reported in [27].

In the northern part of the reserve, there is a wetland that is not directly connected to the river but indirectly influenced by its water level, as well as by rainfall patterns and the human management of the hydrologic resource for agriculture. In the past, the wetland had the typical C-shape of an abandoned river meander [28] but, nowadays, its shape has changed to a more oval form (DP, personal observation). However, despite its shrinkage, this environment hosts native freshwater flora (e.g., *Potamogeton nodosus* Poir) and fauna (e.g., *Rana latastei* Boulenger 1879 and *Unio* spp.).

### 2.2. Target Species

During independent census surveys carried out in the reserve between 2021 and 2023, we recorded the presence of several alien species, including two freshwater macroinvertebrates, namely the red swamp crayfish *Procambarus clarkii* Girard, 1882 and the North American snail *Physella acuta* (Draparnaud, 1805); four fishes, the European bitterling *Rhodeus amaro* Bloch, 1782, the stone moroko *Pseudorasbora parva* Bleeker, 1860, the Pumpkinseed *Lepomis gibbosus* Linnaeus, 1758, and the Eastern mosquitofish *Gambusia holbrooki* Girard, 1859; one reptile with two sub-species, the pond slider *Trachemys scripta* (*T. s. scripta*, Thunberg in Schoepff, 1792 and *T. s. elegans*, Wied-Neuwied, 1839) and one amphibian, the Balkan frog *Pelophylax kurtmuelleri* (Gayda, 1940). Considering that almost all of the mentioned non-native species are listed on the EU list of Union Concern (EU, 2019/1262) and the relevance of this protected area is for the safeguarding of residual floodplain habitats and native (including endemic) species occurring in the wetland, most listed in the Annexes II and IV of the Habitats Directive (e.g., *Triturus carnifex* (Laurenti, 1768), *Rana latastei* (Boulenger, 1879) and Odonata (e.g., *Ophiogomphus cecilia* (Geoffroy in Fourcroy, 1785)), we were immediately aware of the potential threats represented by the non-indigenous species for the native fauna.

### 2.3. Assessment Methods

We applied two different evaluation protocols (INSEAT and NNRM, [16,21]) to provide informative data to address the management decisions for effective conservation actions. Specifically, the INSEAT assessment protocol stated the impacts of invasive alien species (IAS) on 16 ecosystem services, commonly grouped into three extensive categories, namely provisioning, regulating and cultural, based on the ecosystem services framework [21]. This method excluded ecosystem supporting services, assuming that they were included in the other services, and therefore were already counted in the assessment [29]. The protocol, established following the ecosystem services classification from the Millennium Ecosystem Assessment [30,31], required the operator(s) to answer six questions (Q1–Q6). Questions 1 and 2 had a score that varied from 1 to 3, question 3 from 1 to 4 and questions 4 and 5 varied from −4 to 4. Q5 included a list of potential benefits IAS could provide, assuming that possible adverse effects on the environment could be mitigated. Finally, Q6 allowed the evaluator to add comments or additional information to support the assessment, and thus it did not require any scores.

The INSEAT protocol was filled using the available information found in the scientific or grey literature supported eventually by expert judgment. Therefore, for each answer, it was requested to indicate a confidence level for each answer (after the Intergovernmental Panel on Climate Change (IPCC) [32]) as well as the source of the provided information (e.g., scientific, grey literature or personal opinion) to better support the final score. For more details on the methodology, see [16].

The assessed IAS were ranked according to a Likert scale, assuming that a widely distributed IAS should have a greater impact than a narrower distribution one. Combining the scores associated to the answers from Q1 to Q4 of the INSEAT protocol, two different sub-indexes were obtained, namely the General Impact Index and the IAS Manageability Index.

The General Impact index was calculated by multiplying the score obtained in Q4 (species impacts, with a range from −4 to 4) with the score from Q1 (IAS spatial occupation, scores from 1 to 3). The impact index varied from −12 to 12: scores from −12 to −4 indicated strong negative impacts, scores from −4 to 4 indicated mild or null effects and scores from 4 to 12 indicated strong positive effects.

Q4 also provided a detailed list of the ecosystem services on which IAS could have an impact. Assigning a score to each specific impact and multiplying it by the Q1 score gave the score of the impact from IAS on a single ecosystem service.

Question 2 and question 3 were built as a rapid evaluation of the feasibility of eradication. The IAS Manageability Index was calculated by multiplying the score of question 2 (spread capacity, scores from 1 to 3) and the score of question 3 (management effort, scores from 1 to 3).

The NNRM scheme aimed to evaluate the IAS eradication feasibility through a semi-quantitative score. This approach could be applied to any IAS in any area and was based on expert judgement supported by the available information (grey or scientific literature) associated with a confidence level [21,32]. It could be applied even when data on both IAS abundance and distribution are limited, and it satisfies the international requirements for risk management [3,33,34,35,36]. Defining the risk management area and describing the possible invasion scenario was the first step. The scenario should be based on existing or predicted data on the distribution, abundance, and auto-ecology of the evaluated IAS. The second step concerned the choice of a specific eradication strategy or several combined strategies to be assessed, which should still be realistic and likely to eradicate the IAS in the target area. The eradication feasibility was assessed through a protocol based on seven questions and a final expert judgement on the possible strategy, which took into account all the previous scores, which ranged from 1 (the least favourable) to 5 (the most reliable) for each answer (see the supplementary materials in [21] for more details). The overall score was defined through expert judgment, following the approach proposed by the EPPO (The European and Mediterranean Plant Protection Organization) and other risk assessment protocols [37,38].

All the information necessary for the application of both the INSEAT and NNRM tools was collected using the Web of Science and Google Scholar search engines by inserting specific keywords, such as ‘impact’, ‘management’, ‘eradication’, ‘ecosystem services’ and ‘assessment’, followed by the scientific name of the species. Finally, specialised databases such as CABI-Invasive Species and the IUCN Global Invasive Species Database were used to collect ecological and management information.

## 3. Results

**Figure 2:**
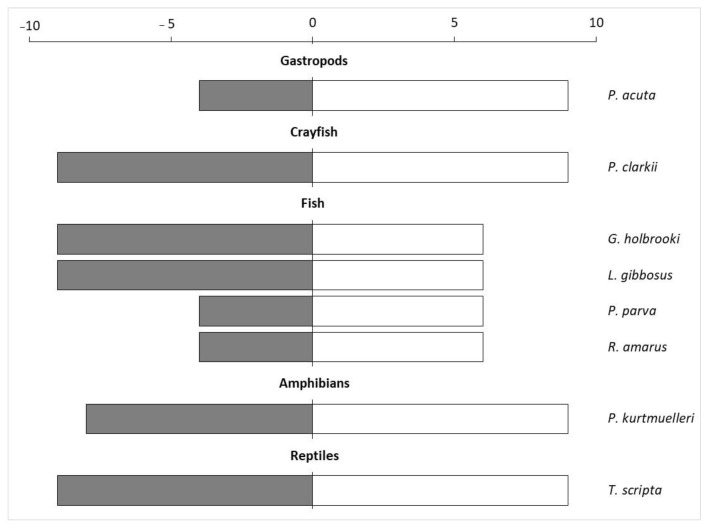
General Impact index (dark grey bars) calculated by multiplying the score obtained in Q1 with the Q4 score, and the Manageability Index (white bars) obtained by multiplying Q2 and Q3 of the eight aquatic species evaluated using the INSEAT protocol.

The application of the INSEAT protocol on the pool of the selected invasive species showed that four of them had the highest General Impact Index, namely *P. clarkii*, *G. holbrooki*, *L. gibbosus* and *T. scripta* (Figure 2).

The spreading capacity was evaluated as “potentially high” for all the evaluated species (Q2), while the management effort (Q3) was assessed as “high” for almost all the species, especially for *P. acuta*, *P. clarkii*, *P. kurtmuelleri* and *T. scripta* (Supplementary Material S1). Overall, the Manageability Index was higher for crayfish, gastropods, amphibians, and reptiles compared to fish (Figure 2).

**Figure 3:**
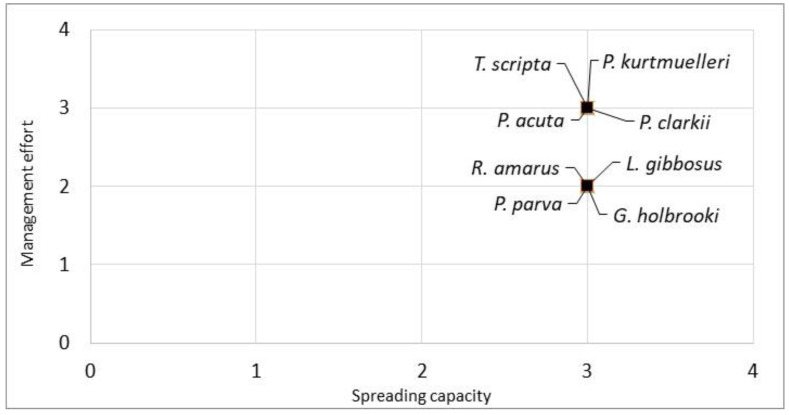
Manageability plot of the eight evaluated aquatic species. The spreading capacity scores (Q2) and the management effort scores (Q3) were derived from the INSEAT protocol. The species in the top right of the graph are the species with a lower manageability.

Furthermore, combining the spreading capacity (Q2) of the evaluated species with the management effort required to contain them (Q3), all the species were clustered in the top right part of the graph, indicating a high spreading capacity and high management effort. The species with the highest combined scores were *P. clarkii*, *P. acuta*, *T. scripta* and *P. kurtmuelleri* (Figure 3).

**Figure 4:**
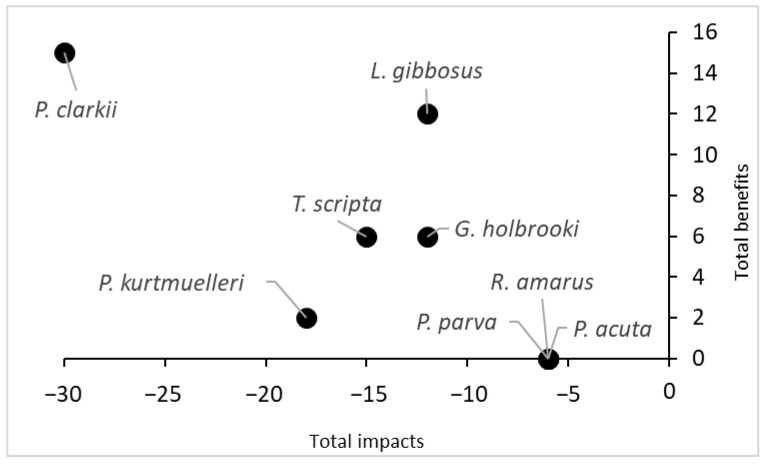
Comparison of the total impacts and total benefits of the eight aquatic species recorded in the protected wetland. The scores were obtained from Q4 and Q5 of the INSEAT protocol.

Finally, considering the total score obtained by summing the scores of the negative impacts and the scores of the potential benefits, *P. clarkii* turned out to be the species with the highest impact on the ecosystem, but it also had the highest potential benefit (Figure 4). Conversely, three species (*R. amarus*, *P. parva* and *P. acuta*) did not show any potential benefits and had low impact scores.

Once the impacts and potential benefits were assessed for each aquatic alien species detected in the integral natural reserve, we reviewed and evaluated the different eradication techniques available for each target species using the Non-Native Risk Management scheme (NNRM). From our literature review, it turned out that crayfish had the most diverse eradication techniques (N = 7), followed by fish (N = 5), gastropods (N = 3), reptiles (N = 2) and finally amphibians (N = 1) (Table 1).

**Figure 5:**
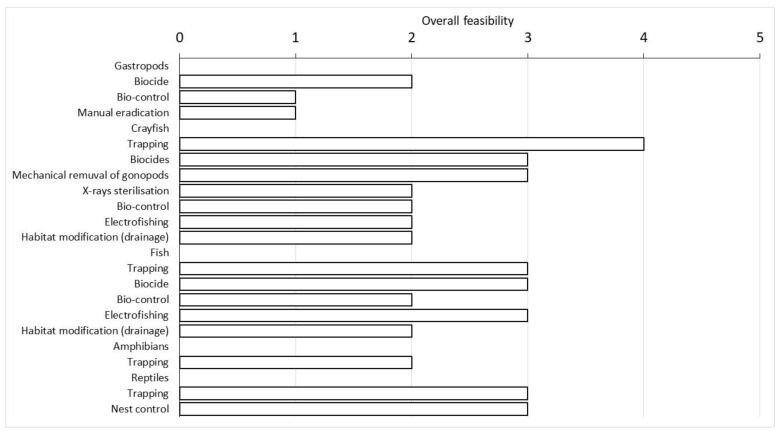
Overall feasibility score of the eradication techniques evaluated using the NNRM scheme divided for each group of alien species reported in the wetland. For more details on each evaluated technique, see Supplementary Materials S2.

Indeed, trapping was the most feasible technique for *P. clarkii*, followed by the application of specific biocides and the mechanical removal of male gonopods. The first two techniques could also be used to eradicate all fish species and, together with electrofishing, were evaluated as the most feasible. For *P. acuta*, the most suitable eradication technique was the use of biocides, which appeared effective, although with a low level of feasibility in the management area (Table 1; Figure 5).

*Trachemys scripta* was mainly eradicated by trapping, but its overall feasibility was not considered as high as crayfish. An alternative practice included nest control, which achieved the same overall score as trapping (Figure 5). Trapping was also the only technique currently available for the eradication of *P. kurtmuelleri*, but its overall feasibility received a rather low score (Figure 5).

## 4. Discussion

The evaluation obtained using the General Impacts index indicated that *P. clarkii*, *L. gibbosus*, *G. holbrooki* and *T. scripta* had higher impacts on ecosystem services. These four aquatic alien species were also the most abundant in the area (Q1) and they were assessed with a higher ecosystem impact score (Q4) (Supplementary Material S1). Moreover, their impact on native biodiversity is well known in scientific literature and most of them are classified as “harmful” according to the EICAT classification proposed by the IUCN [38]. Our results agreed with such an evaluation, confirming the worrying status of those species in the wetland of the integral natural reserve Bosco Siro Negri.

Overall, all the target species caused strong pressure on local fauna and flora, mainly due to their predator activity and their competition for food and space [39,40,41,42]. At least in one case (*P. kurtmuelleri*), there was also another kind of impact on the local fauna, i.e., hybridization, inducing reproductive parasitism and the local extinction of native frogs [43].

Another aspect to consider in the assessment of the impacts of the target alien species was the fact that at least some of them acted as disease vectors. A case in point was *P. clarkii*, which hosted pathogens or parasites that are sometimes even dangerous for human health [44], or *P. kurtmuelleri*, which may be a carrier of the chytrid fungus *Batrachochytrium dendrobatidis* capable of inducing mass amphibian mortality worldwide through chytridiomycosis [45]. In addition, *P. clarkii* also had an impact on regulating services, such as sediment and water quality, and also caused damages to banks (see Supplementary Materials S1). Finally, it is worth noting that all the evaluated species shared a similar impact on cultural services, leading to a homogenization of local biodiversity (see Supplementary Materials S1).

Given the role of integral natural reserves for preserving local biodiversity, our assessment was based on the effect that alien species could induce a loss of ecological and conservation interest in the area. Nevertheless, the magnitude of the impacts turned out to be related to the extent of alien species’ distribution in the assessed area (Supplementary Materials S1). Conversely, in the literature, there were examples that demonstrated the direct and/or indirect positive effects of alien species on ecosystem services [4]. Thus, it is important to consider the balance between the impacts and potential benefits [46]. In our study case, only a few species showed relatively potential positive effects on ecosystem services. For instance, despite the high level of impacts, *P. clarkii* represented a resource in terms of food provisions for animals, humans and the local economy (as bait, e.g., [4]) due to its abundance in the area (Figure 4). However, as its impacts outweighed its benefits, this species was listed in the EU list of Union Concern, and thus any use is forbidden by law. Other species, such as *T. scripta* and *L. gibbosus*, could have potential benefits as other services because they could be considered as pets (i.e., aquarium trade), or regarding *G. affinis*, they could be used for the biological control of pests. However, due to the restrictions imposed by EU laws on alien species, these options are now quite remote.

Our census suggested that all the alien species assessed in this study were well established in the area and had a high spreading capacity, resulting in a high level of manageability and costs, which meant that the effort to manage these species was generally challenging (Figure 3). Nevertheless, considering the characteristics of the natural reserve (i.e., a closed environment of relatively big dimensions and its classification as an integral natural state reserve), it is theoretically possible to manage the eight aquatic alien species with the available techniques.

Among the eight alien species investigated in this study, *P. clarkii* had the highest number of eradication techniques (N = 7). Crayfish are among the most invasive freshwater species, and many large-scale projects have been carried out worldwide to contain their invasion using different techniques (i.e., the CRAYNET project, the EU Life Claw projeclaw.eu). Conversely, the species with the lowest number of available eradication techniques was the Balkan frog *P. kurtmuelleri* (N = 1), for which no species-specific methodologies have been tested. For this reason, in this study, we referred to the available eradication procedures (i.e., trapping/removal) that were already successfully tested for other invasive anurans (i.e., *Rana catesbeiana* [47]).

As the Balkan frog can hybridise with native water frogs in the wild [44], its eradication can be realistic only at the very first stage of the invasion process, ideally prior to or at the very beginning of the first reproductive season, as the removal strategy becomes more and more unrealistic as the breeding season proceeds. Moreover, as the alien cannot be distinguished with high confidence from native water frogs by morphology alone, the removal strategy must be supported by molecular identification.

Generally, trapping was considered the most feasible eradication technique among those reviewed for all the species considered, with the only exception of *P. acuta* (Table 1). Indeed, this technique was the most unexpensive one and it often appeared feasible in terms of its effectiveness, practicality, acceptability, and costs (i.e., equipment and personnel) (Supplementary Material S2).

The use of biocides appeared suitable for crayfish, fish and gastropods, according to the overall feasibility of this technique. However, this method was not the best solution for managing alien species within a protected area, as it also negatively impacted non-target species. The use of biocides also had a low level of acceptability due to its possible negative effects, and therefore could be considered reliable only for *P. acuta* (Supplementary Material S2).

Electrofishing was another technique proposed as a useful alternative to eradicate more than one alien species. It appeared reliable for fish, although it was strongly dependent on the type of environment. For example, our managed area had a soft bottom (i.e., mud, sand and vegetation debris) that affected the efficiency of the technique, as the electricity could be dispersed by the surrounding environment and capture could also be hindered by vegetation.

Another option was the drainage of the wetland. This technique has been applied in other cases with some positive results for fish [48] and less positive results for crayfish, which can survive outside the water for quite a long time [49]. For the other species, this technique was not taken into consideration because it was not considered feasible and due the lack of evidence in the literature.

Biocontrol (by predator and parasites) was also proposed to eradicate crayfish, fish and gastropods [50,51]. This technique received a good score, especially for crayfish and fish, as it appeared to be effective and was relatively inexpensive and highly acceptable. On the other hand, caution must be used while introducing new predators/parasites in the environment as they could potentially target new prey and hosts [51].

The species-specific eradication techniques were X-ray sterilisation and the mechanical removal of male gonopods (crayfish, [52,53,54], manual eradication (gastropods, [55]) and nest control (reptiles, [56]). The first two options (X-ray sterilisation and the mechanical removal of male gonopods) appeared to be highly acceptable as they did not have a negative impact on local biodiversity. They were effective and they have been performed in the past, but with partial success [52,53,54]. However, they have also been assessed with a low feasibility for various reasons. X-ray sterilisation requires an appropriate structure for the X-ray and the resources to stabilise the animals for the required time, with a plausible increase in cost. Regarding the manual removal of *P. acuta*, this technique has been successfully applied to a similar species (*Pomacea insularum*, [55]), but considering the spatial extension of the wetland, the small size of the species and the difficulties in finding its eggs, this strategy was evaluated as very ineffective. Finally, nest control for *T. scripta* was proposed as useful in small and clearly visible areas of nesting sites [56]. It requires minimal equipment consisting of a small hoe, gloves and containers for the eggs and juveniles, and it has a low impact on the aquatic environment and its biota. However, due to the occurrence of native species (*Emys orbicularis*), this technique must be carried out by expert staff [56,57].

To be truly effective, most of the eradication techniques listed here should be applied in combination to achieve the definitive eradication of as many as possible alien species occurring in the area. Nonetheless, the techniques applied depend not only on the benefits in terms of biological feasibility, but also the side effects on non-target species and costs [58]. The positive ecological effects of invasive species eradication are often overestimated, while the costs are understated [59]. In addition, considering the high reproductive rate and spread ability of many alien species, reinvasion of the same area could be very likely, so the prevention of alien species release appears far more feasible both from ecological and cost perspectives. Finally, it is also mandatory to take into consideration the acceptability of the eradication techniques for the stakeholders, as it could represent a central issue for effective management of IAS [60].

The conservation of natural reserves is fundamental to the protection of local biodiversity, and the management of alien species in these areas is a major challenge for managers and researchers [3,61]. Similarly, riverine wetlands could be considered as stepping-stone habitats where freshwater species survive in the midst of anthropogenic pressures, as stated by the Ramsar Convention (1971).

## 5. Conclusions

Protected areas should be constantly monitored for possible biological invasions, promoting the prevention and control of the vectors and pathways of introduction. The discovery of eight different alien species belonging to very different taxa and impacting the ecosystem at several levels of complexity was worrying, and it raised concerns on the management protocols of invasive species in protected areas.

Considering the environmental characteristics of the studied area (relatively small dimensions, absence of an inlet or outlet, and the fact that the wetland was not connected to other wetlands), the currently available eradication techniques and the ecological characteristics of each target species, we were aware that IAS would be hard to completely eradicate, as most of them were already well established. In conclusion, the study of biological invasion in protected areas and the application of management assessment tools represent unique research opportunities for improving our knowledge of more effective management techniques and performing practical conservation actions (i.e., trapping for crayfish, nests control for turtles, biocontrol using native species for crayfish and fish) to reduce—at least to some extent—the population density of the target species and the negative impacts on local biodiversity and mitigate the effects of non-native species on ecosystem services.

## Figures and Tables

**Table 1:** Results of the application of the Non-Native Risk Management scheme (NNRM) on the eradication techniques reported in the literature. The response score was a five-point scale from 1 to 5, where 1 indicates the least favourable and 5 the most. N.A. indicates that no information was available. The overall score and its level of confidence was defined through expert judgment: high level (H) or medium level (M) of confidence. The overall eradication feasibility score was estimated by taking into consideration all the collected information and expressing a judgement on each eradication technique; it is not calculated as the mean of other scores.

Eradication Strategy/Risk Management	Effectiveness	Praticality	Costs	Impacts	Acceptability	Window of Opportunity	Likelihood of Reinvasion	Overall Feasibility	Overall Confidence
Gastropods (*P. acuta*)									
Biocide	3	4	N.A.	2	4	1	1	2	H
Biocontrol (predators)	1	3	N.A.	2	2	1	1	1	H
Manual eradication	1	1	N.A.	5	5	1	1	1	H
Crayfish (*P. clarkii*)									
Trapping	4	5	5	5	5	3	3	4	H
Chemical/biocides	4	3	5	2	4	3	3	3	H
Mechanical removal of gonopods	3	3	5	5	5	3	3	3	H
X-rays sterilisation	4	2	N.A.	5	5	3	3	2	H
Biocontrol	3	3	5	2	5	3	3	2	H
Electro fishing	2	3	5	2	4	3	3	2	H
Habitat modification (drainage)	2	1	N.A.	1	2	3	3	2	H
Fish (*L. gibbosus*; *P. parva*; *R. amaro*)									
Trapping	4	4	5	5	5	3	2	3	H
Biocide	4	3	5	2	3	3	2	3	H
Biocontrol (genetic)	4	3	N.A.	3	3	3	2	2	M
Electro fishing	3	3	5	2	4	3	2	3	H
Habitat modification (drainage)	5	1	N.A.	1	2	3	2	2	H
Amphibians (*P. kurtmuelleri*)									
Trapping/removal	3	4	4	4	4	2	2	2	M
Reptiles (*T. scripta*)									
Trapping	4	4	4	5	5	3	2	3	H
Nest control	3	4	5	4	4	3	2	3	H

## Data Availability

All the information is provided in the Supplementary Materials.

## Supplementary Materials

The following supporting information can be downloaded at: https://www.mdpi.com/article/10.3390/biology13010064/s1, Supplementary Material S1: Application of the Invasive Species Effects Assessment Tool; Supplementary Material S2: Application of the Non-native Risk Management Assessment (NNRM).
