# Supplementary material for: Impacts, Potential Benefits and Eradication Feasibility of Aquatic Alien Species in an Integral Natural State Reserve
Source: Biology (Basel). 2024 Jan 22;13(1):64. doi: 10.3390/biology13010064 (PMC10813597; doi:10.3390/biology13010064)
Supplement: Supplementary file 1 [file biology-13-00064-s001.zip › Supplementary Materials 2_Paganelli et al.pdf]

Supplementary Materials S2

Impacts, potential benefits and eradication feasibility of the aquatic alien species in an integral natural state reserve

Application of the Non-native Risk Management Assessment (NNRM). Answers are highlighted in yellow

Risk management area:	Integral natural state reserve BOSCO NEGRI
Objective:	eradication
Organism name:	*Procambarus clarkii*
Assessor name(s):	Daniele Paganelli
Date / version:	20-1-23

**Title**	**Response**	**Confidence**	**Justification**
1. Define the scenario	*Well established in the wild with high abundance. The risk assessment area is almost a closed environment, with no freshwater inlet or outlet*		
2. Define the eradication strategy	Trapping		
3a. How effective is the strategy?	5 - V EFFECTIVE<br>4 – EFFECTIVE<br>3 – MODERATE<br>2 – INEFFECTIVE<br>1 - V INEFFECTIVE	3 – HIGH<br>2 – MED<br>1 – LOW	Trapping strategy could be effective but only in a closed environment (Bills and Marking, 1988; Hein et al., 2007; Kreig et al., 2020)
3b. How practical is the strategy?	5 - V PRACTICAL<br>4 – PRACTICAL<br>3 – MODERATE<br>2 – IMPRACTICAL<br>1 – V IMPRACTICAL	3 – HIGH<br>2 – MED<br>1 – LOW	In the assessment area there are not particular barriers to create problems in applying this strategy
3c. How expensive is the strategy?	5 (<£50K)<br>4 (£50-200K)<br>3 (£200K-1M)<br>2 (1-10M)<br>1 (> £10M)	3 – HIGH<br>2 – MED<br>1 – LOW	The strategy costs include: two operators for 3 hours a day, 4 times per month; bait cost (2 tin of cat food for 10 traps, twice a month) and the costs of the traps (at the beginning of the activity).
3d. How much negative impact would the strategy have?	5 – MINIMAL<br>4 – MINOR<br>3 – MODERATE<br>2 – MAJOR<br>1 – MASSIVE	3 – HIGH<br>2 – MED<br>1 – LOW	Traps are quite selective for the assessed species and, moreover, leaving them in the water for 24 hours reduce the likelihood to catch non-target species
3e. How acceptable is the strategy?	5 - V ACCEPTABLE<br>4 – ACCEPTABLE<br>3 – MODERATE<br>2 – UNACCEPTABLE<br>1 - V UNACCEPTABLE	3 – HIGH<br>2 – MED<br>1 – LOW	Crayfish are not considered as pet species by stakeholders, so it is unlikely that their eradication is not considered acceptable

4. What is the window of opportunity for implementing the strategy?	5 (10+ YRS)<br>4 (4-10 YRS)<br>3 (1 – 3 YRS)<br>2 (2 MTHS - 1 YR)<br>1 (< 2 MTHS)	3 – HIGH<br>2 – MED<br>1 – LOW	The reproduction rate of this species is very high and moreover it could have a rapid expansion rate because it is very mobile (CABI.org)
5. What is the likelihood of reinvasion?	5 – V UNLIKELY<br>4 – UNLIKELY<br>3 – MODERATE<br>2 – LIKELY<br>1 – V LIKELY	3 – HIGH<br>2 – MED<br>1 – LOW	The assessment area is basically a closed pond, but it is very close to the main river and it is surrounded by the secondary hydrographic system which is invaded by the species
6. Conclusion (overall feasibility of eradication)	5 – V HIGH<br>4 – HIGH<br>3 – MEDIUM<br>2 – LOW<br>1 – V LOW	3 – HIGH<br>2 – MED<br>1 – LOW	This strategy is quite effective if there are specific environmental conditions

Risk management area:	Integral natural state reserve BOSCO NEGRI
Objective:	eradication
Organism name:	*Procambarus clarkii*
Assessor name(s):	Daniele Paganelli
Date / version:	23-1-23

**Title**	**Response**	**Confidence**	**Justification**
1. Define the scenario	*The species is well established in the wild with high abundance. The risk assessment area is almost a closed environment, with no freshwater inlet or outlet*		
2. Define the eradication strategy	Chemical/biocides		
3a. How effective is the strategy?	5 - V EFFECTIVE<br>4 – EFFECTIVE<br>3 – MODERATE<br>2 – INEFFECTIVE<br>1 - V INEFFECTIVE	3 – HIGH<br>2 – MED<br>1 – LOW	Various studies confirmed the use of pyrethrum or other biocides is a suitable technique to eradicate crayfish. However, it should be associated with other action such as habitat modification and control strategies (Peay et al., 2019)
3b. How practical is the strategy?	5 - V PRACTICAL<br>4 – PRACTICAL<br>3 – MODERATE<br>2 – IMPRACTICAL<br>1 – V IMPRACTICAL	3 – HIGH<br>2 – MED<br>1 – LOW	In order to use this strategy, it is necessary to use a small boat which is not easy to take in this freshwater environment because it is surrounded by woods, moreover it should be necessary to check/treat all the different habitats in the oxbow lake
3c. How expensive is the strategy?	5 (<£50K)<br>4 (£50-200K)<br>3 (£200K-1M)	3 – HIGH<br>2 – MED<br>1 – LOW	Considering the comparison with other techniques, the costs of the

	2 (1-10M)<br>1 (> £10M)		use of biocides is considered quite low (Manfrin et al., 2019)
3d. How much negative impact would the strategy have?	5 – MINIMAL<br>4 – MINOR<br>3 – MODERATE<br>2 – MAJOR<br>1 – MASSIVE	3 – HIGH<br>2 – MED<br>1 – LOW	Because of the environmental characteristics, it is necessary to use a high concentration of biocides and this could affect fish, amphibians and reptiles (Peay et al., 2019)
3e. How acceptable is the strategy?	5 - V ACCEPTABLE<br>4 – ACCEPTABLE<br>3 – MODERATE<br>2 – UNACCEPTABLE<br>1 - V UNACCEPTABLE	3 – HIGH<br>2 – MED<br>1 – LOW	Crayfish are not considered as pet species by stakeholders, so it is unlikely that their eradication is not considered acceptable. Moreover, various studies indicates that the reaction of the main stakeholders was positive but only after being reassured on the potential problems caused using biocides (Peay et al., 2019)
4. What is the window of opportunity for implementing the strategy?	5 (10+ YRS)<br>4 (4-10 YRS)<br>3 (1 – 3 YRS)<br>2 (2 MTHS - 1 YR)<br>1 (< 2 MTHS)	3 – HIGH<br>2 – MED<br>1 – LOW	The reproduction rate of this species is very high and moreover it could have a rapid expansion rate because it is very mobile (CABI.org)
5. What is the likelihood of reinvasion?	5 – V UNLIKELY<br>4 – UNLIKELY<br>3 – MODERATE<br>2 – LIKELY<br>1 – V LIKELY	3 – HIGH<br>2 – MED<br>1 – LOW	The assessment area is basically a closed pond, but it is very close to the main river and it is surrounded by the secondary hydrographic system which is invaded by the species
6. Conclusion (overall feasibility of eradication)	5 – V HIGH<br>4 – HIGH<br>3 – MEDIUM<br>2 – LOW<br>1 – V LOW	3 – HIGH<br>2 – MED<br>1 – LOW	This technique has been very effective in other environments and it could be effective even in this assessment area but it should be integrated with other techniques

Risk management area:	Integral natural state reserve BOSCO NEGRI
Objective:	eradication
Organism name:	*Procambarus clarkii*
Assessor name(s):	Daniele Paganelli
Date / version:	23-1-23

**Title**	**Response**	**Confidence**	**Justification**
1. Define the scenario	*The species is well established in the wild with high abundance. The risk assessment area is almost a closed environment, with no freshwater inlet or outlet*		
2. Define the eradication strategy	Mechanical removal of gonopods		

3a. How effective is the strategy?	5 - V EFFECTIVE<br>4 – EFFECTIVE<br>3 – MODERATE<br>2 – INEFFECTIVE<br>1 - V INEFFECTIVE	3 – HIGH<br>2 – MED<br>1 – LOW	Various studies confirmed the use of this technique could be effective to eradicate crayfish, mainly in small and closed environment. However, it is important to consider the behaviour of the crayfish that could be altered and their moulting capacity (Johovic et al., 2019).
3b. How practical is the strategy?	5 - V PRACTICAL<br>4 – PRACTICAL<br>3 – MODERATE<br>2 – IMPRACTICAL<br>1 – V IMPRACTICAL	3 – HIGH<br>2 – MED<br>1 – LOW	This strategy could present some difficulties in its application because specimens should be sterilised in the field (Stebbing et al., 2014)
3c. How expensive is the strategy?	5 (<£50K)<br>4 (£50-200K)<br>3 (£200K-1M)<br>2 (1-10M)<br>1 (> £10M)	3 – HIGH<br>2 – MED<br>1 – LOW	Direct costs are not available in literature but they could be compared with the trapping one
3d. How much negative impact would the strategy have?	5 – MINIMAL<br>4 – MINOR<br>3 – MODERATE<br>2 – MAJOR<br>1 – MASSIVE	3 – HIGH<br>2 – MED<br>1 – LOW	This technique doesn't have any collateral impacts on the biodiversity and human health (Johovic et al., 2019)
3e. How acceptable is the strategy?	5 - V ACCEPTABLE<br>4 – ACCEPTABLE<br>3 – MODERATE<br>2 – UNACCEPTABLE<br>1 - V UNACCEPTABLE	3 – HIGH<br>2 – MED<br>1 – LOW	Crayfish are not considered as pet species by stakeholders, so it is unlikely that their eradication is not considered acceptable
4. What is the window of opportunity for implementing the strategy?	5 (10+ YRS)<br>4 (4-10 YRS)<br>3 (1 – 3 YRS)<br>2 (2 MTHS - 1 YR)<br>1 (< 2 MTHS)	3 – HIGH<br>2 – MED<br>1 – LOW	The reproduction rate of this species is very high and moreover it could have a rapid expansion rate because it is very mobile (CABI.org)
5. What is the likelihood of reinvasion?	5 – V UNLIKELY<br>4 – UNLIKELY<br>3 – MODERATE<br>2 – LIKELY<br>1 – V LIKELY	3 – HIGH<br>2 – MED<br>1 – LOW	The assessment area is basically a closed pond, but it is very close to the main river and it is surrounded by the secondary hydrographic system which is invaded by the species

6. Conclusion (overall feasibility of eradication)	5 – V HIGH<br>4 – HIGH<br>3 – MEDIUM<br>2 – LOW<br>1 – V LOW	3 – HIGH<br>2 – MED<br>1 – LOW	Overall, this technique could be feasible because the oxbow lake is a closed environment and the dimension of the population is not big. However, considering the crayfish behaviour, their physiology (moulting), and the quite low practicality (specimens sterilisation in the field) this technique alone is not the best solution for the eradication of the species

Risk management area:	Integral natural state reserve BOSCO NEGRI
Objective:	eradication
Organism name:	*Procambarus clarkii*
Assessor name(s):	Daniele Paganelli
Date / version:	23-1-23

**Title**	**Response**	**Confidence**	**Justification**
1. Define the scenario	*The species is well established in the wild with high abundance. The risk assessment area is almost a closed environment, with no freshwater inlet or outlet*		
2. Define the eradication strategy	Sterile males with X-ray		
3a. How effective is the strategy?	5 - V EFFECTIVE<br>4 – EFFECTIVE<br>3 – MODERATE<br>2 – INEFFECTIVE<br>1 - V INEFFECTIVE	3 – HIGH<br>2 – MED<br>1 – LOW	Various studies confirmed the use of this technique is effective to eradicate crayfish. However, it should be associated with other action such as trapping to achieve the final eradication (Piazza et al., 2015; Giglio et al., 2018; Aquiloni et al., 2014)
3b. How practical is the strategy?	5 - V PRACTICAL<br>4 – PRACTICAL<br>3 – MODERATE<br>2 – IMPRACTICAL<br>1 – V IMPRACTICAL	3 – HIGH<br>2 – MED<br>1 – LOW	This strategy could be quite impractical because it implies the finding of an available structure for the X-ray, as well as the resources for stabulating the specimens before and after the treatment

3c. How expensive is the strategy?	5 (<£50K)<br>4 (£50-200K)<br>3 (£200K-1M)<br>2 (1-10M)<br>1 (> £10M)	3 – HIGH<br>2 – MED<br>1 – LOW	Costs of the use of X ray are not available in literature
3d. How much negative impact would the strategy have?	5 – MINIMAL<br>4 – MINOR<br>3 – MODERATE<br>2 – MAJOR<br>1 – MASSIVE	3 – HIGH<br>2 – MED<br>1 – LOW	This technique doesn't have any collateral impacts on the biodiversity and human health (Piazza et al., 2015; Manfrin et al., 2019)
3e. How acceptable is the strategy?	5 - V ACCEPTABLE<br>4 – ACCEPTABLE<br>3 – MODERATE<br>2 – UNACCEPTABLE<br>1 - V UNACCEPTABLE	3 – HIGH<br>2 – MED<br>1 – LOW	Crayfish are not considered as pet species by stakeholders, so it is unlikely that their eradication is not considered acceptable
4. What is the window of opportunity for implementing the strategy?	5 (10+ YRS)<br>4 (4-10 YRS)<br>3 (1 – 3 YRS)<br>2 (2 MTHS - 1 YR)<br>1 (< 2 MTHS)	3 – HIGH<br>2 – MED<br>1 – LOW	The reproduction rate of this species is very high and moreover it could have a rapid expansion rate because it is very mobile (CABI.org)
5. What is the likelihood of reinvasion?	5 – V UNLIKELY<br>4 – UNLIKELY<br>3 – MODERATE<br>2 – LIKELY<br>1 – V LIKELY	3 – HIGH<br>2 – MED<br>1 – LOW	The assessment area is basically a closed pond, but it is very close to the main river and it is surrounded by the secondary hydrographic system which is invaded by the species
6. Conclusion (overall feasibility of eradication)	5 – V HIGH<br>4 – HIGH<br>3 – MEDIUM<br>2 – LOW<br>1 – V LOW	3 – HIGH<br>2 – MED<br>1 – LOW	This technique could be considered very effective but it is not so easy to apply because various reasons: first it is necessary to find a structure available for the X-ray treatment, then the stabulation of the specimens requires space and time, finally costs are not easy to quantify.

Risk management area:	Integral natural state reserve BOSCO NEGRI
Objective:	eradication
Organism name:	*Procambarus clarkii*
Assessor name(s):	Daniele Paganelli
Date / version:	23-1-23

Title	Response	Confidence	Justification
1. Define the scenario	*The species is well established in the wild with high abundance. The risk assessment area is almost a closed environment, with no freshwater inlet or outlet*		
2. Define the eradication strategy	Biological control (predators and pathogens)		
3a. How effective is the strategy?	5 - V EFFECTIVE<br>4 – EFFECTIVE<br>3 – MODERATE<br>2 – INEFFECTIVE<br>1 - V INEFFECTIVE	3 – HIGH<br>2 – MED<br>1 – LOW	In literature, there are examples of bio-control projects (introduction of pikes, eels, pathogens) but none of them seem to have acted efficiently (Elvira et al., 1996; Aquiloni et al., 2010; Hein et al., 2007; Stebbing et al., 2014; Longshaw et al., 2012)
3b. How practical is the strategy?	5 - V PRACTICAL<br>4 – PRACTICAL<br>3 – MODERATE<br>2 – IMPRACTICAL<br>1 – V IMPRACTICAL	3 – HIGH<br>2 – MED<br>1 – LOW	The introduction of predators (e.g. pikes) could be relatively easy because the oxbow lake represents the natural habitat for this fish
3c. How expensive is the strategy?	5 (<£50K)<br>4 (£50-200K)<br>3 (£200K-1M)<br>2 (1-10M)<br>1 (> £10M)	3 – HIGH<br>2 – MED<br>1 – LOW	Direct costs are not available in literature
3d. How much negative impact would the strategy have?	5 – MINIMAL<br>4 – MINOR<br>3 – MODERATE<br>2 – MAJOR<br>1 – MASSIVE	3 – HIGH<br>2 – MED<br>1 – LOW	Introductions of predators may not be environmentally sustainable and they represent new pressures on the already invaded ecosystems (Manfrin et al., 2019)
3e. How acceptable is the strategy?	5 - V ACCEPTABLE<br>4 – ACCEPTABLE<br>3 – MODERATE<br>2 – UNACCEPTABLE<br>1 - V UNACCEPTABLE	3 – HIGH<br>2 – MED<br>1 – LOW	Crayfish are not considered as pet species by stakeholders, so it is unlikely that their eradication is not considered acceptable
4. What is the window of opportunity for implementing the strategy?	5 (10+ YRS)<br>4 (4-10 YRS)<br>3 (1 – 3 YRS)<br>2 (2 MTHS - 1 YR)<br>1 (< 2 MTHS)	3 – HIGH<br>2 – MED<br>1 – LOW	The reproduction rate of this species is very high and moreover it could have a rapid expansion rate because it is very mobile (CABI.org)

5. What is the likelihood of reinvasion?	5 – V UNLIKELY<br>4 – UNLIKELY<br>3 – MODERATE<br>2 – LIKELY<br>1 – V LIKELY	3 – HIGH<br>2 – MED<br>1 – LOW	The assessment area is basically a closed pond, but it is very close to the main river and it is surrounded by the secondary hydrographic system which is invaded by the species
6. Conclusion (overall feasibility of eradication)	5 – V HIGH<br>4 – HIGH<br>3 – MEDIUM<br>2 – LOW<br>1 – V LOW	3 – HIGH<br>2 – MED<br>1 – LOW	Introducing a top predator in such a small habitat could become a problem for the ecosystem: once the predator has been introduced, it needs preys to survive. Moreover, it is important to introduce native species.<br>The introduction of pathogens could be a risk because we must be sure that it is species-specific

Risk management area:	Integral natural state reserve BOSCO NEGRI
Objective:	eradication
Organism name:	*Procambarus clarkii*
Assessor name(s):	Daniele Paganelli
Date / version:	23-1-23

Title	Response	Confidence	Justification
1. Define the scenario	*Well established in the wild with high abundance. The risk assessment area is almost a closed environment, with no freshwater inlet or outlet*		
2. Define the eradication strategy	Electro fishing		
3a. How effective is the strategy?	5 - V EFFECTIVE<br>4 – EFFECTIVE<br>3 – MODERATE<br>2 – INEFFECTIVE<br>1 - V INEFFECTIVE	3 – HIGH<br>2 – MED<br>1 – LOW	Despite there are examples of successful eradication in scientific literature (Kreig et al., 2020), this strategy was used in different freshwater environment. An oxbow lake has soft bottom (i.e., mud, sand and vegetation debris) so the electricity is not so effective because it could be dispersed by the surrounding environment and the capture of the crayfish could be interfered by the vegetation and wooden debris
3b. How practical is the strategy?	5 - V PRACTICAL<br>4 – PRACTICAL<br>3 – MODERATE	3 – HIGH<br>2 – MED<br>1 – LOW	In order to use this strategy, it is necessary to use a small boat which is not easy to take in this freshwater

	2 – IMPRACTICAL<br>1 – V IMPRACTICAL		environment because it is surrounded by woods
3c. How expensive is the strategy?	5 (<£50K)<br>4 (£50-200K)<br>3 (£200K-1M)<br>2 (1-10M)<br>1 (> £10M)	3 – HIGH<br>2 – MED<br>1 – LOW	The main costs are related to the purchase of the electro fisher and the operators (two at least)
3d. How much negative impact would the strategy have?	5 – MINIMAL<br>4 – MINOR<br>3 – MODERATE<br>2 – MAJOR<br>1 – MASSIVE	3 – HIGH<br>2 – MED<br>1 – LOW	Because of the environmental characteristics, it is necessary to use a high voltage electricity and it causes damages to the rest of the fauna
3e. How acceptable is the strategy?	5 - V ACCEPTABLE<br>4 – ACCEPTABLE<br>3 – MODERATE<br>2 – UNACCEPTABLE<br>1 - V UNACCEPTABLE	3 – HIGH<br>2 – MED<br>1 – LOW	Crayfish are not considered as pet species by stakeholders, so it is unlikely that their eradication is not considered acceptable
4. What is the window of opportunity for implementing the strategy?	5 (10+ YRS)<br>4 (4-10 YRS)<br>3 (1 – 3 YRS)<br>2 (2 MTHS - 1 YR)<br>1 (< 2 MTHS)	3 – HIGH<br>2 – MED<br>1 – LOW	The reproduction rate of this species is very high and moreover it could have a rapid expansion rate because it is very mobile (CABI.org)
5. What is the likelihood of reinvasion?	5 – V UNLIKELY<br>4 – UNLIKELY<br>3 – MODERATE<br>2 – LIKELY<br>1 – V LIKELY	3 – HIGH<br>2 – MED<br>1 – LOW	The assessment area is basically a closed pond, but it is very closed to the main river and it is surrounded by the secondary hydrographic system which is invaded by the species
6. Conclusion (overall feasibility of eradication)	5 – V HIGH<br>4 – HIGH<br>3 – MEDIUM<br>2 – LOW<br>1 – V LOW	3 – HIGH<br>2 – MED<br>1 – LOW	Despite this technique could be effective in other environments, overall, it is not so effective in this assessment area

Risk management area:	Integral natural state reserve BOSCO NEGRI
Objective:	eradication
Organism name:	*Procambarus clarkii*
Assessor name(s):	Daniele Paganelli
Date / version:	23-1-23

**Title**	**Response**	**Confidence**	**Justification**
1. Define the scenario	*The species is well established in the wild with high abundance. The risk assessment area is almost a closed environment, with no freshwater inlet or outlet*		
2. Define the eradication strategy	Habitat modification (drainage)		

3a. How effective is the strategy?	5 - V EFFECTIVE<br>4 – EFFECTIVE<br>3 – MODERATE<br>2 – INEFFECTIVE<br>1 - V INEFFECTIVE	3 – HIGH<br>2 – MED<br>1 – LOW	The effectiveness of this technique is quite low because crayfish (especially *P. clarkii*) can survive for a long time out of the water due to its burrowing activity (Manfrin et al., 2019; Gherardi et al., 2011; Kerby et al., 2005; Holdich and Reeve, 1991)
3b. How practical is the strategy?	5 - V PRACTICAL<br>4 – PRACTICAL<br>3 – MODERATE<br>2 – IMPRACTICAL<br>1 – V IMPRACTICAL	3 – HIGH<br>2 – MED<br>1 – LOW	In the contest of the risk management area, this technique is not practical from a naturalistic (i.e., remove of all the non-target species) point of view but also from a technical point of view
3c. How expensive is the strategy?	5 (<£50K)<br>4 (£50-200K)<br>3 (£200K-1M)<br>2 (1-10M)<br>1 (> £10M)	3 – HIGH<br>2 – MED<br>1 – LOW	Direct costs are not available in literature
3d. How much negative impact would the strategy have?	5 – MINIMAL<br>4 – MINOR<br>3 – MODERATE<br>2 – MAJOR<br>1 – MASSIVE	3 – HIGH<br>2 – MED<br>1 – LOW	The drainage of the oxbow lake causes high impacts on non-target species
3e. How acceptable is the strategy?	5 - V ACCEPTABLE<br>4 – ACCEPTABLE<br>3 – MODERATE<br>2 – UNACCEPTABLE<br>1 - V UNACCEPTABLE	3 – HIGH<br>2 – MED<br>1 – LOW	Crayfish are not considered as pet species by stakeholders, so it is unlikely that their eradication is not considered acceptable. However, the alteration of a such delicate ecosystem could cause other problems and thus this technique is not considered acceptable
4. What is the window of opportunity for implementing the strategy?	5 (10+ YRS)<br>4 (4-10 YRS)<br>3 (1 – 3 YRS)<br>2 (2 MTHS - 1 YR)<br>1 (< 2 MTHS)	3 – HIGH<br>2 – MED<br>1 – LOW	The reproduction rate of this species is very high and moreover it could have a rapid expansion rate because it is very mobile (CABI.org)
5. What is the likelihood of reinvasion?	5 – V UNLIKELY<br>4 – UNLIKELY<br>3 – MODERATE<br>2 – LIKELY<br>1 – V LIKELY	3 – HIGH<br>2 – MED<br>1 – LOW	The assessment area is basically a closed pond, but it is very close to the main river and it is surrounded by the secondary hydrographic system which is invaded by the species

6. Conclusion (overall feasibility of eradication)	5 – V HIGH<br>4 – HIGH<br>3 – MEDIUM<br>2 – LOW<br>1 – V LOW	3 – HIGH<br>2 – MED<br>1 – LOW	The overall feasibility of this technique is low because of the impacts, the costs and the effectiveness. The oxbow lake is subjected to strong fluctuations of the water level related to the level of the closed Ticino river, so it is not possible to appreciate the effects of a natural drainage on crayfish population. However, it is possible to see the effects of drainage, using trapping once the water level would have increased

Risk management area:	Integral natural state reserve BOSCO NEGRI
Objective:	eradication
Organism name:	Fish (*Rhodeus amaro; Pseudorasbora parva; Lepomis gibbosus; Gambusia holbrooki)*
Assessor name(s):	Daniele Paganelli
Date / version:	04-05-23

**Title**	**Response**	**Confidence**	**Justification**
1. Define the scenario	*All the evaluated species are well established in the wild with high abundance. The risk management area is almost a closed environment, with no freshwater inlet or outlet*		
2. Define the eradication strategy	Trapping (Traps and hand net)		
3a. How effective is the strategy?	5 - V EFFECTIVE<br>4 – EFFECTIVE<br>3 – MODERATE<br>2 – INEFFECTIVE<br>1 - V INEFFECTIVE	3 – HIGH<br>2 – MED<br>1 – LOW	This technique works in small systems with low initial densities of small-sized target species. It could also be used in bigger environments, but it should be adapted (Ruiz et al., 2013)
3b. How practical is the strategy?	5 - V PRACTICAL<br>4 – PRACTICAL<br>3 – MODERATE<br>2 – IMPRACTICAL<br>1 – V IMPRACTICAL	3 – HIGH<br>2 – MED<br>1 – LOW	It is a quite practical techniques that does not require specific equipment and it is easy to perform
3c. How expensive is the strategy?	5 (<£50K)<br>4 (£50-200K)<br>3 (£200K-1M)<br>2 (1-10M)<br>1 (> £10M)	3 – HIGH<br>2 – MED<br>1 – LOW	Direct costs are not available in literature but, considering the material used, they should be quite low
3d. How much negative impact would the strategy have?	5 – MINIMAL<br>4 – MINOR<br>3 – MODERATE<br>2 – MAJOR<br>1 – MASSIVE	3 – HIGH<br>2 – MED<br>1 – LOW	This technique has low impacts on the aquatic environment and its biota

3e. How acceptable is the strategy?	5 - V ACCEPTABLE<br>4 – ACCEPTABLE<br>3 – MODERATE<br>2 – UNACCEPTABLE<br>1 - V UNACCEPTABLE	3 – HIGH<br>2 – MED<br>1 – LOW	Considering that it does not have high negative impacts and the target fish are not considered of particular interest, using this technique should not cause any problems
4. What is the window of opportunity for implementing the strategy?	5 (10+ YRS)<br>4 (4-10 YRS)<br>3 (1 – 3 YRS)<br>2 (2 MTHS - 1 YR)<br>1 (< 2 MTHS)	3 – HIGH<br>2 – MED<br>1 – LOW	These species have a high reproduction rate
5. What is the likelihood of reinvasion?	5 – V UNLIKELY<br>4 – UNLIKELY<br>3 – MODERATE<br>2 – LIKELY<br>1 – V LIKELY	3 – HIGH<br>2 – MED<br>1 – LOW	These species are very well spread in the surrounding area and thus the likelihood of reinvasion of the wetland is quite high
6. Conclusion (overall feasibility of eradication)	5 – V HIGH<br>4 – HIGH<br>3 – MEDIUM<br>2 – LOW<br>1 – V LOW	3 – HIGH<br>2 – MED<br>1 – LOW	Because of the environmental characteristics of the managed wetland, overall, this technique could be suitable to eradicate alien fish

Risk management area:	Integral natural state reserve BOSCO NEGRI
Objective:	eradication
Organism name:	Fish (*Rhodeus amaro; Pseudorasbora parva; Lepomis gibbosus; Gambusia holbrooki)*
Assessor name(s):	Daniele Paganelli
Date / version:	04-05-23

**Title**	**Response**	**Confidence**	**Justification**
1. Define the scenario	*All the evaluated species are well established in the wild with high abundance. The risk management area is almost a closed environment, with no freshwater inlet or outlet*		
2. Define the eradication strategy	Biocides		
3a. How effective is the strategy?	5 - V EFFECTIVE<br>4 – EFFECTIVE<br>3 – MODERATE<br>2 – INEFFECTIVE<br>1 - V INEFFECTIVE	3 – HIGH<br>2 – MED<br>1 – LOW	The piscicide 'rotenone' has been applied successfully for eradication of *Pseudorasbora parva* in relatively small water bodies in the UK (Britton et al., 2010)
3b. How practical is the strategy?	5 - V PRACTICAL<br>4 – PRACTICAL<br>3 – MODERATE<br>2 – IMPRACTICAL<br>1 – V IMPRACTICAL	3 – HIGH<br>2 – MED<br>1 – LOW	It implies the remove of the native fauna and, eventually, its repopulation
3c. How expensive is the strategy?	5 (<£50K)<br>4 (£50-200K)	3 – HIGH<br>2 – MED	The costs of the use of biocide is not particularly high (e.g. cost of

	3 (£200K-1M)<br>2 (1-10M)<br>1 (> £10M)	1 – LOW	rotenone only GBP20·L^{-1}; in 2017 ≈€20·L^{-1}); however, the quantity of chemicals depends on the character of the water body and the species to eradicate (Britton et al., 2010). However, it requires intensive manpower input during key stages (Britton and Brazier, 2006). Some species require a high concentration of rotenone, some simply a longer duration of exposure (e.g. *Pseudorasbora parva* requires 2× the exposure of most other cyprinid fishes to be killed by rotenone; see Allen et al., 2006)
3d. How much negative impact would the strategy have?	5 – MINIMAL<br>4 – MINOR<br>3 – MODERATE<br>2 – MAJOR<br>1 – MASSIVE	3 – HIGH<br>2 – MED<br>1 – LOW	Rotenone kills all fish species and is also harmful to amphibians and aquatic invertebrates, so collateral damage is high. However, if it used in specific dosage and periods of the year, its impact could be lower
3e. How acceptable is the strategy?	5 - V ACCEPTABLE<br>4 – ACCEPTABLE<br>3 – MODERATE<br>2 – UNACCEPTABLE<br>1 - V UNACCEPTABLE	3 – HIGH<br>2 – MED<br>1 – LOW	It is very likely that the general public and/or stakeholders may show resistance to the approach (on environmental / animal welfare grounds), though this may be possible to overcome through awareness and education of the general public regarding the risks to biodiversity, the environment and ecosystem services posed by invasive non-native species (Bremner and Park, 2007)
4. What is the window of opportunity for implementing the strategy?	5 (10+ YRS)<br>4 (4-10 YRS)<br>3 (1 – 3 YRS)<br>2 (2 MTHS - 1 YR)<br>1 (< 2 MTHS)	3 – HIGH<br>2 – MED<br>1 – LOW	These species have a high rate of reproduction
5. What is the likelihood of reinvasion?	5 – V UNLIKELY<br>4 – UNLIKELY<br>3 – MODERATE<br>2 – LIKELY<br>1 – V LIKELY	3 – HIGH<br>2 – MED<br>1 – LOW	These species are very well spread in the surrounding area and thus the likelihood of reinvasion of the wetland is quite high
6. Conclusion (overall feasibility of eradication)	5 – V HIGH<br>4 – HIGH<br>3 – MEDIUM<br>2 – LOW<br>1 – V LOW	3 – HIGH<br>2 – MED<br>1 – LOW	Overall, this technique could be considered quite valid for the eradication of the alien fish. However, its impacts on the

			biodiversity could represent an obstacle for its application. Moreover, the use of some biocides (e.g. rotenone) is not acceptable in several EU-countries

Risk management area:	Integral natural state reserve BOSCO NEGRI
Objective:	eradication
Organism name:	Fish *(Rhodeus amaro; Pseudorasbora parva; Lepomis gibbosus; Gambusia holbrooki)*
Assessor name(s):	Daniele Paganelli
Date / version:	23-1-23

**Title**	**Response**	**Confidence**	**Justification**
1. Define the scenario	*All the evaluated species are well established in the wild with high abundance. The risk management area is almost a closed environment, with no freshwater inlet or outlet*		
2. Define the eradication strategy	Biological control (predators)		
3a. How effective is the strategy?	5 - V EFFECTIVE<br>4 – EFFECTIVE<br>3 – MODERATE<br>2 – INEFFECTIVE<br>1 - V INEFFECTIVE	3 – HIGH<br>2 – MED<br>1 – LOW	There are examples of the success of this technique and other examle where it did not work (Bajer et al., 2019; Rytwinski et al., 2019).
3b. How practical is the strategy?	5 - V PRACTICAL<br>4 – PRACTICAL<br>3 – MODERATE<br>2 – IMPRACTICAL<br>1 – V IMPRACTICAL	3 – HIGH<br>2 – MED<br>1 – LOW	Introduce fish specimens in this wetland could be relatively difficult because of the difficulties in reaching the water.
3c. How expensive is the strategy?	5 (<£50K)<br>4 (£50-200K)<br>3 (£200K-1M)<br>2 (1-10M)<br>1 (> £10M)	3 – HIGH<br>2 – MED<br>1 – LOW	Direct costs are not available in literature
3d. How much negative impact would the strategy have?	5 – MINIMAL<br>4 – MINOR<br>3 – MODERATE<br>2 – MAJOR<br>1 – MASSIVE	3 – HIGH<br>2 – MED<br>1 – LOW	The introduction of new predators in a such small area could alterate the trophic chain and the equilibrium of the ecosystem
3e. How acceptable is the strategy?	5 - V ACCEPTABLE<br>4 – ACCEPTABLE<br>3 – MODERATE<br>2 – UNACCEPTABLE	3 – HIGH<br>2 – MED<br>1 – LOW	This strategy has a very high level of accettability because it uses native species and the target species do not have any socio-economic interest

	1 - V UNACCEPTABLE		
4. What is the window of opportunity for implementing the strategy?	5 (10+ YRS)<br>4 (4-10 YRS)<br>3 (1 – 3 YRS)<br>2 (2 MTHS - 1 YR)<br>1 (< 2 MTHS)	3 – HIGH<br>2 – MED<br>1 – LOW	These species have a high reproduction rate
5. What is the likelihood of reinvasion?	5 – V UNLIKELY<br>4 – UNLIKELY<br>3 – MODERATE<br>2 – LIKELY<br>1 – V LIKELY	3 – HIGH<br>2 – MED<br>1 – LOW	These species are very well spread in the surrounding area and thus the likelihood of reinvasion of the wetland is quite high
6. Conclusion (overall feasibility of eradication)	5 – V HIGH<br>4 – HIGH<br>3 – MEDIUM<br>2 – LOW<br>1 – V LOW	3 – HIGH<br>2 – MED<br>1 – LOW	This technique could be effective but the overall feasibility has been considered low because the introduction of new predators could alterate the equilibrium of the ecosystem.<br>In our opinion, a combination of different techniques could lead to more effecttive eradication result.

Risk management area:	Integral natural state reserve BOSCO NEGRI
Objective:	eradication
Organism name:	Fish *(Rhodeus amaro; Pseudorasbora parva; Lepomis gibbosus; Gambusia holbrooki)*
Assessor name(s):	Daniele Paganelli
Date / version:	04-05-23

**Title**	**Response**	**Confidence**	**Justification**
1. Define the scenario	*All the evaluated species are well established in the wild with high abundance. The risk management area is almost a closed environment, with no freshwater inlet or outlet*		
2. Define the eradication strategy	Electro fishing		
3a. How effective is the strategy?	5 - V EFFECTIVE<br>4 – EFFECTIVE<br>3 – MODERATE<br>2 – INEFFECTIVE<br>1 - V INEFFECTIVE	3 – HIGH<br>2 – MED<br>1 – LOW	This technique is suitable for catching fish but, considering how it works and its technical characteristics, its effectiveness for the eradication purpose is not

			always very effective especially in this environment.<br>- Fisk, J. M. II, C. W. Morgeson, and M. E. Polera. 2019. Evaluation of recreational hand-crank electrofishing on introduced catfish species in Southeastern North Carolina. North American Journal of Fisheries Management 39:150–165<br>- Peterson, Douglas P., et al. "When eradication is not an option: modeling strategies for electrofishing suppression of nonnative brook trout to foster persistence of sympatric native cutthroat trout in small streams." North American Journal of Fisheries Management 28.6 (2008): 1847-1867.<br>- Day, Casey C., et al. "Evaluation of management factors affecting the relative success of a brook trout eradication program using YY male fish and electrofishing suppression." Canadian Journal of Fisheries and Aquatic Sciences 78.8 (2021): 1109-1119.
3b. How practical is the strategy?	5 - V PRACTICAL<br>4 – PRACTICAL<br>3 – MODERATE<br>2 – IMPRACTICAL<br>1 – V IMPRACTICAL	3 – HIGH<br>2 – MED<br>1 – LOW	In order to use this strategy, it is necessary to use a small boat which is not easy to take in this freshwater environment because it is surrounded by woods
3c. How expensive is the strategy?	5 (<£50K)<br>4 (£50-200K)<br>3 (£200K-1M)<br>2 (1-10M)<br>1 (> £10M)	3 – HIGH<br>2 – MED<br>1 – LOW	The main costs are related to the purchase of the electro fisher and the operators (two at least) Verreycken and Copp, 2017. Study on Invasive Alien Species –

			Development of risk assessments to tackle priority species and enhance prevention
3d. How much negative impact would the strategy have?	5 – MINIMAL<br>4 – MINOR<br>3 – MODERATE<br>2 – MAJOR<br>1 – MASSIVE	3 – HIGH<br>2 – MED<br>1 – LOW	Because of the environmental characteristics, it is necessary to use a high voltage electricity and it causes damages to the rest of the fauna
3e. How acceptable is the strategy?	5 - V ACCEPTABLE<br>4 – ACCEPTABLE<br>3 – MODERATE<br>2 – UNACCEPTABLE<br>1 - V UNACCEPTABLE	3 – HIGH<br>2 – MED<br>1 – LOW	The eradication of alien fish is not always seen as acceptable. However, this resistance is more likely for big species which are suitable for angling recreation activity
4. What is the window of opportunity for implementing the strategy?	5 (10+ YRS)<br>4 (4-10 YRS)<br>3 (1 – 3 YRS)<br>2 (2 MTHS - 1 YR)<br>1 (< 2 MTHS)	3 – HIGH<br>2 – MED<br>1 – LOW	These species have a high rate of reproduction
5. What is the likelihood of reinvasion?	5 – V UNLIKELY<br>4 – UNLIKELY<br>3 – MODERATE<br>2 – LIKELY<br>1 – V LIKELY	3 – HIGH<br>2 – MED<br>1 – LOW	These species are very well spread in the surrounding area and thus the likelihood of reinvasion of the wetland is quite high
6. Conclusion (overall feasibility of eradication)	5 – V HIGH<br>4 – HIGH<br>3 – MEDIUM<br>2 – LOW<br>1 – V LOW	3 – HIGH<br>2 – MED<br>1 – LOW	Despite this technique could be effective in other environments, overall, it is not so effective in this management area

Risk management area:	Integral natural state reserve BOSCO NEGRI
Objective:	eradication
Organism name:	Fish *(Rhodeus amaro; Pseudorasbora parva; Lepomis gibbosus; Gambusia holbrooki)*
Assessor name(s):	Daniele Paganelli
Date / version:	04-05-23

**Title**	**Response**	**Confidence**	**Justification**
1. Define the scenario	*All the evaluated species are well established in the wild with high abundance. The risk management area is almost a closed environment, with no freshwater inlet or outlet*		
2. Define the eradication strategy	Drainage		
3a. How effective is the strategy?	5 - V EFFECTIVE<br>4 – EFFECTIVE<br>3 – MODERATE<br>2 – INEFFECTIVE	3 – HIGH<br>2 – MED<br>1 – LOW	This technique appears to be effective for all the invasive fish. There are examples with *Gambusia*

	1 - V INEFFECTIVE		spp. and *Pseudorasbora parva* (Lemmens et al. 2015).
3b. How practical is the strategy?	5 - V PRACTICAL<br>4 – PRACTICAL<br>3 – MODERATE<br>2 – IMPRACTICAL<br>1 – V IMPRACTICAL	3 – HIGH<br>2 – MED<br>1 – LOW	In the contest of the risk management area, this technique is not practical from a naturalistic (i.e. remove of all the non-target species) point of view but also from a technical point of view
3c. How expensive is the strategy?	5 (<£50K)<br>4 (£50-200K)<br>3 (£200K-1M)<br>2 (1-10M)<br>1 (> £10M)	3 – HIGH<br>2 – MED<br>1 – LOW	Direct costs are not available in literature
3d. How much negative impact would the strategy have?	5 – MINIMAL<br>4 – MINOR<br>3 – MODERATE<br>2 – MAJOR<br>1 – MASSIVE	3 – HIGH<br>2 – MED<br>1 – LOW	The drainage of the oxbow lake causes high impacts on non-target species. In areas where native amphibians are present, drainage should be performed between September and January (after metamorphosis of the amphibian larvae and before the start of the new breeding season), that it avoids or minimises the risks of non-target organisms (invertebrates, amphibians) being affected by the work (Verreycken and Copp, 2016).
3e. How acceptable is the strategy?	5 - V ACCEPTABLE<br>4 – ACCEPTABLE<br>3 – MODERATE<br>2 – UNACCEPTABLE<br>1 - V UNACCEPTABLE	3 – HIGH<br>2 – MED<br>1 – LOW	It is very likely that the general public and/or stakeholders may show resistance to the approach (on environmental / animal welfare grounds), though this may be possible to overcome through awareness and education of the general public regarding the risks to biodiversity, the environment and ecosystem services posed by invasive non-native species (Bremner and Park, 2007).
4. What is the window of opportunity for implementing the strategy?	5 (10+ YRS)<br>4 (4-10 YRS)<br>3 (1 – 3 YRS)<br>2 (2 MTHS - 1 YR)<br>1 (< 2 MTHS)	3 – HIGH<br>2 – MED<br>1 – LOW	These species have a high reproduction rate
5. What is the likelihood of reinvasion?	5 – V UNLIKELY<br>4 – UNLIKELY<br>3 – MODERATE<br>2 – LIKELY<br>1 – V LIKELY	3 – HIGH<br>2 – MED<br>1 – LOW	These species are very well spread in the surrounding area and thus the likelihood of reinvasion of the wetland is quite high

6. Conclusion (overall feasibility of eradication)	5 – V HIGH<br>4 – HIGH<br>3 – MEDIUM<br>2 – LOW<br>1 – V LOW	3 – HIGH<br>2 – MED<br>1 – LOW	The overall feasibility of this technique is low because of the impacts, the costs and the effectiveness. The oxbow lake is subjected to strong fluctuations of the water level related to the level of the closed river Ticino so it is not possible to appreciate the effects of a natural drainage on the fish population.

Risk management area:	Integral natural state reserve BOSCO NEGRI
Objective:	eradication
Organism name:	Gastropods (*Physella acuta*)
Assessor name(s):	Daniele Paganelli
Date / version:	11-05-23

**Title**	**Response**	**Confidence**	**Justification**
1. Define the scenario	*The evaluated species is well established in the wild with high abundance. The risk management area is almost a closed environment, with no freshwater inlet or outlet*		
2. Define the eradication strategy	Biocides		
3a. How effective is the strategy?	5 - V EFFECTIVE<br>4 – EFFECTIVE<br>3 – MODERATE<br>2 – INEFFECTIVE<br>1 - V INEFFECTIVE	3 – HIGH<br>2 – MED<br>1 – LOW	Several research papers suggested that the use of biocides could work in the control of gastropods species, but they also stated that this technique works when the molluscs density is low and, to be effective, it should be necessary to use high dosage of the chemical compounds (Martin et al., 2012; Müller et al., 2015; David et al., 2020)
3b. How practical is the strategy?	5 - V PRACTICAL<br>4 – PRACTICAL<br>3 – MODERATE<br>2 – IMPRACTICAL<br>1 – V IMPRACTICAL	3 – HIGH<br>2 – MED<br>1 – LOW	The use of biocides does not require specific tools
3c. How expensive is the strategy?	5 (<£50K)<br>4 (£50-200K)<br>3 (£200K-1M)<br>2 (1-10M)<br>1 (> £10M)	3 – HIGH<br>2 – MED<br>1 – LOW	No information about costs is available in literature
3d. How much negative impact would the strategy have?	5 – MINIMAL<br>4 – MINOR<br>3 – MODERATE<br>2 – MAJOR<br>1 – MASSIVE	3 – HIGH<br>2 – MED<br>1 – LOW	According to Okete (2015), Niclosamid (Baylucide, Bayer Germany) is the only commercially available synthetic molluscicides applied on a large scale (WHO,

			2004). However, this synthetic molluscicide tends to be generally biocidal, toxic to fish and microscopic aquatic animals at lower concentrations than those required to kill the snails and affecting many of the plants in the snail habitat.
3e. How acceptable is the strategy?	5 - V ACCEPTABLE<br>4 – ACCEPTABLE<br>3 – MODERATE<br>2 – UNACCEPTABLE<br>1 - V UNACCEPTABLE	3 – HIGH<br>2 – MED<br>1 – LOW	This technique could be acceptable only if does not have any impacts on local biodiversity
4. What is the window of opportunity for implementing the strategy?	5 (10+ YRS)<br>4 (4-10 YRS)<br>3 (1 – 3 YRS)<br>2 (2 MTHS - 1 YR)<br>1 (< 2 MTHS)	3 – HIGH<br>2 – MED<br>1 – LOW	There are examples in literature where after an initial positive effect of this technique (Raines, 2009), live snails were found a week after the treatment (Raines, 2009)
5. What is the likelihood of reinvasion?	5 – V UNLIKELY<br>4 – UNLIKELY<br>3 – MODERATE<br>2 – LIKELY<br>1 – V LIKELY	3 – HIGH<br>2 – MED<br>1 – LOW	Considering the environmental characteristic of the wetland and the bio-ecological traits of the invasive species, the reinvasion is very likely
6. Conclusion (overall feasibility of eradication)	5 – V HIGH<br>4 – HIGH<br>3 – MEDIUM<br>2 – LOW<br>1 – V LOW	3 – HIGH<br>2 – MED<br>1 – LOW	Overall, this technique could be useful to contrast invasive molluscs but its application has problems related its impacts on local biodiversity and the biological traits of the molluscs increase the likelihood of reinvasion

Risk management area:	Integral natural state reserve BOSCO NEGRI
Objective:	eradication
Organism name:	Gastropods (*Physella acuta*)
Assessor name(s):	Daniele Paganelli
Date / version:	12-05-23

**Title**	**Response**	**Confidence**	**Justification**
1. Define the scenario	*The evaluated species is well established in the wild with high abundance. The risk management area is almost a closed environment, with no freshwater inlet or outlet*		
2. Define the eradication strategy	Fish predation		
3a. How effective is the strategy?	5 - V EFFECTIVE<br>4 – EFFECTIVE<br>3 – MODERATE	3 – HIGH<br>2 – MED<br>1 – LOW	One of the possible fish predators is *L. gibbosus* which is already present in the wetland. Despite its presence,

	2 – INEFFECTIVE<br>1 - V INEFFECTIVE		we recorded high abundance of *P. acuta*, meaning that this technique is not effective. In literature, there are other examples of the ineffectively of this technique (Martin et al., 2012)
3b. How practical is the strategy?	5 - V PRACTICAL<br>4 – PRACTICAL<br>3 – MODERATE<br>2 – IMPRACTICAL<br>1 – V IMPRACTICAL	3 – HIGH<br>2 – MED<br>1 – LOW	The introduction of predators could be moderately practical
3c. How expensive is the strategy?	5 (<£50K)<br>4 (£50-200K)<br>3 (£200K-1M)<br>2 (1-10M)<br>1 (> £10M)	3 – HIGH<br>2 – MED<br>1 – LOW	No information about costs is available in literature
3d. How much negative impact would the strategy have?	5 – MINIMAL<br>4 – MINOR<br>3 – MODERATE<br>2 – MAJOR<br>1 – MASSIVE	3 – HIGH<br>2 – MED<br>1 – LOW	The introduction of potential predators could alterate the ecosystem. Especially, the increase of the abundance of L. gibbosus could cause more problems
3e. How acceptable is the strategy?	5 - V ACCEPTABLE<br>4 – ACCEPTABLE<br>3 – MODERATE<br>2 – UNACCEPTABLE<br>1 - V UNACCEPTABLE	3 – HIGH<br>2 – MED<br>1 – LOW	The increasing of the abundance of an invasive species already present in the wetland, could cause more problems and alterate the equilibrium of the ecosystem. Because of that, this strategy is not acceptable
4. What is the window of opportunity for implementing the strategy?	5 (10+ YRS)<br>4 (4-10 YRS)<br>3 (1 – 3 YRS)<br>2 (2 MTHS - 1 YR)<br>1 (< 2 MTHS)	3 – HIGH<br>2 – MED<br>1 – LOW	Considering the high reproduction rate of the species, this technique is not suitable
5. What is the likelihood of reinvasion?	5 – V UNLIKELY<br>4 – UNLIKELY<br>3 – MODERATE<br>2 – LIKELY<br>1 – V LIKELY	3 – HIGH<br>2 – MED<br>1 – LOW	Considering the environmental characteristic of the wetland and the bio-ecological traits of the invasive species, the reinvasion is very likely
6. Conclusion (overall feasibility of eradication)	5 – V HIGH<br>4 – HIGH<br>3 – MEDIUM<br>2 – LOW<br>1 – V LOW	3 – HIGH<br>2 – MED<br>1 – LOW	Overall, this technique should not be considered feasible because of the impacts listed above

Risk management area:	Integral natural state reserve BOSCO NEGRI
Objective:	eradication
Organism name:	Gastropods (*Physella acuta*)

Assessor name(s):	Daniele Paganelli
Date / version:	11-05-23

**Title**	**Response**	**Confidence**	**Justification**
1. Define the scenario	*The evaluated species is well established in the wild with high abundance. The risk management area is almost a closed environment, with no freshwater inlet or outlet*		
2. Define the eradication strategy	Manual eradication		
3a. How effective is the strategy?	5 - V EFFECTIVE<br>4 – EFFECTIVE<br>3 – MODERATE<br>2 – INEFFECTIVE<br>1 - V INEFFECTIVE	3 – HIGH<br>2 – MED<br>1 – LOW	Considering the dimension of the assessed species and the difficulties to find the eggs, this strategy has been evaluated as very ineffective
3b. How practical is the strategy?	5 - V PRACTICAL<br>4 – PRACTICAL<br>3 – MODERATE<br>2 – IMPRACTICAL<br>1 – V IMPRACTICAL	3 – HIGH<br>2 – MED<br>1 – LOW	Despite there are some examples of this eradication technique on other mollusc species (e.g. Apple Snail *Pomacea insularum*, Martin et al., 2012), this strategy could not considered practical for the assessed invasive species
3c. How expensive is the strategy?	5 (<£50K)<br>4 (£50-200K)<br>3 (£200K-1M)<br>2 (1-10M)<br>1 (> £10M)	3 – HIGH<br>2 – MED<br>1 – LOW	No information about costs is available in literature
3d. How much negative impact would the strategy have?	5 – MINIMAL<br>4 – MINOR<br>3 – MODERATE<br>2 – MAJOR<br>1 – MASSIVE	3 – HIGH<br>2 – MED<br>1 – LOW	This technique does not have any other negative impacts
3e. How acceptable is the strategy?	5 - V ACCEPTABLE<br>4 – ACCEPTABLE<br>3 – MODERATE<br>2 – UNACCEPTABLE<br>1 - V UNACCEPTABLE	3 – HIGH<br>2 – MED<br>1 – LOW	This technique does not have any impacts on other species so it could be considered very acceptable
4. What is the window of opportunity for implementing the strategy?	5 (10+ YRS)<br>4 (4-10 YRS)<br>3 (1 – 3 YRS)<br>2 (2 MTHS - 1 YR)<br>1 (< 2 MTHS)	3 – HIGH<br>2 – MED<br>1 – LOW	Considering the high reproduction rate of the species, this technique should be repeated very often on order to be effective
5. What is the likelihood of reinvasion?	5 – V UNLIKELY<br>4 – UNLIKELY<br>3 – MODERATE<br>2 – LIKELY<br>1 – V LIKELY	3 – HIGH<br>2 – MED<br>1 – LOW	Considering the environmental characteristic of the wetland and the bio-ecological traits of the invasive species, the reinvasion is very likely

6. Conclusion (overall feasibility of eradication)	5 – V HIGH<br>4 – HIGH<br>3 – MEDIUM<br>2 – LOW<br>1 – V LOW	3 – HIGH<br>2 – MED<br>1 – LOW	Overall, this technique should not be considered feasible because the effort to manually eradicate this species is too high and not effective

Risk management area:	Integral natural state reserve BOSCO NEGRI
Objective:	eradication
Organism name:	Pond slider (*Trachemys scripta* spp.)
Assessor name(s):	Daniele Pellitteri-Rosa
Date / version:	23-06-23

**Title**	**Response**	**Confidence**	**Justification**
1. Define the scenario	*Well established in the wild with high abundance. The risk assessment area is almost a closed environment, with connection to freshwater inlet or outlet (Ticino river)*		
2. Define the eradication strategy	Trapping		
3a. How effective is the strategy?	5 - V EFFECTIVE<br>4 – EFFECTIVE<br>3 – MODERATE<br>2 – INEFFECTIVE<br>1 - V INEFFECTIVE	3 – HIGH<br>2 – MED<br>1 – LOW	The trapping strategy could quite effective but only if it is applied in a closed environment (Gili, 2018)
3b. How practical is the strategy?	5 - V PRACTICAL<br>4 – PRACTICAL<br>3 – MODERATE<br>2 – IMPRACTICAL<br>1 – V IMPRACTICAL	3 – HIGH<br>2 – MED<br>1 – LOW	It is a set of quite practical techniques that require specific equipment (double coil fish traps with guide nets; atoll traps; floating pots with trigger) and it is enough easy to perform (Sancho Alcayde et al., 2015)
3c. How expensive is the strategy?	5 (<£50K)<br>4 (£50-200K)<br>3 (£200K-1M)<br>2 (1-10M)<br>1 (> £10M)	3 – HIGH<br>2 – MED<br>1 – LOW	Direct costs are available in literature (Sancho Alcayde et al., 2015) and could be sustainable if used in a closed and not too broad environment
3d. How much negative impact would the strategy have?	5 – MINIMAL<br>4 – MINOR<br>3 – MODERATE<br>2 – MAJOR<br>1 – MASSIVE	3 – HIGH<br>2 – MED<br>1 – LOW	This technique has low impacts on the aquatic environment and its biota. Animals that might be accidentally caught can be released into the water immediately (Macchi et al., 2020)
3e. How acceptable is the strategy?	5 - V ACCEPTABLE<br>4 – ACCEPTABLE<br>3 – MODERATE<br>2 – UNACCEPTABLE<br>1 - V UNACCEPTABLE	3 – HIGH<br>2 – MED<br>1 – LOW	This strategy is the most used and universally accepted since it does not have high negative impacts and the target freshwater turtles are not considered of particular interest, so this technique should not cause any problems

4. What is the window of opportunity for implementing the strategy?	5 (10+ YRS)<br>4 (4-10 YRS)<br>3 (1 – 3 YRS)<br>2 (2 MTHS - 1 YR)<br>1 (< 2 MTHS)	3 – HIGH<br>2 – MED<br>1 – LOW	These species have a high reproduction rate, lay numerous eggs, with a high reproductive success (Cadi & Joly, 2004)
5. What is the likelihood of reinvasion?	5 – V UNLIKELY<br>4 – UNLIKELY<br>3 – MODERATE<br>2 – LIKELY<br>1 – V LIKELY	3 – HIGH<br>2 – MED<br>1 – LOW	These species are very well spread in the surrounding area and thus the likelihood of reinvasion of the wetland is quite high
6. Conclusion (overall feasibility of eradication)	5 – V HIGH<br>4 – HIGH<br>3 – MEDIUM<br>2 – LOW<br>1 – V LOW	3 – HIGH<br>2 – MED<br>1 – LOW	Because of the environmental characteristics of the managed wetland, overall, this technique could be suitable to eradicate alien freshwater turtles

Risk management area:	Integral natural state reserve BOSCO NEGRI
Objective:	eradication
Organism name:	Pond slider (*Trachemys scripta* spp.)
Assessor name(s):	Daniele Pellitteri-Rosa
Date / version:	23-06-23

**Title**	**Response**	**Confidence**	**Justification**
1. Define the scenario	*Well established in the wild with high abundance. The risk assessment area is almost a closed environment, with connection to freshwater inlet or outlet (Ticino river)*		
2. Define the eradication strategy	Nest control		
3a. How effective is the strategy?	5 - V EFFECTIVE<br>4 – EFFECTIVE<br>3 – MODERATE<br>2 – INEFFECTIVE<br>1 - V INEFFECTIVE	3 – HIGH<br>2 – MED<br>1 – LOW	This strategy could be useful in small and clearly visible areas for nesting sites (Gili, 2018)
3b. How practical is the strategy?	5 - V PRACTICAL<br>4 – PRACTICAL<br>3 – MODERATE<br>2 – IMPRACTICAL<br>1 – V IMPRACTICAL	3 – HIGH<br>2 – MED<br>1 – LOW	It consists in tracking and locating by sight the nests of terrapins near humid areas (Ferri, 2019). The best results in terms of location of the nests are obtained by direct observation by experienced personnel (Sancho Alcayde et al., 2015)
3c. How expensive is the strategy?	5 (<£50K)<br>4 (£50-200K)<br>3 (£200K-1M)<br>2 (1-10M)<br>1 (> £10M)	3 – HIGH<br>2 – MED<br>1 – LOW	It is only necessary to have a minimum equipment consisting of a small hoe, gloves and containers for eggs and juveniles (Sancho Alcayde et al., 2015)

3d. How much negative impact would the strategy have?	5 – MINIMAL<br>4 – MINOR<br>3 – MODERATE<br>2 – MAJOR<br>1 – MASSIVE	3 – HIGH<br>2 – MED<br>1 – LOW	This technique has low impacts on the aquatic environment and its biota. However, since the similarities with the native species, this technique should only be adopted by experienced people (Sancho Alcayde et al., 2015)
3e. How acceptable is the strategy?	5 - V ACCEPTABLE<br>4 – ACCEPTABLE<br>3 – MODERATE<br>2 – UNACCEPTABLE<br>1 - V UNACCEPTABLE	3 – HIGH<br>2 – MED<br>1 – LOW	The strategy has not been frequently adopted in the eradication projects reported in the literature, however returned interesting results where applied, also by using trained dogs (Genovesi, 2009; Gili, 2018)
4. What is the window of opportunity for implementing the strategy?	5 (10+ YRS)<br>4 (4-10 YRS)<br>3 (1 – 3 YRS)<br>2 (2 MTHS - 1 YR)<br>1 (< 2 MTHS)	3 – HIGH<br>2 – MED<br>1 – LOW	These species have a high reproduction rate, lay numerous eggs, with a high reproductive success (Cadi & Joly, 2004)
5. What is the likelihood of reinvasion?	5 – V UNLIKELY<br>4 – UNLIKELY<br>3 – MODERATE<br>2 – LIKELY<br>1 – V LIKELY	3 – HIGH<br>2 – MED<br>1 – LOW	These species are very well spread in the surrounding area and thus the likelihood of reinvasion of the wetland is quite high
6. Conclusion (overall feasibility of eradication)	5 – V HIGH<br>4 – HIGH<br>3 – MEDIUM<br>2 – LOW<br>1 – V LOW	3 – HIGH<br>2 – MED<br>1 – LOW	Because of the environmental characteristics of the managed wetland, overall this strategy could be suitable to eradicate alien freshwater turtles if associated with other trapping techniques

Risk management area:	Integral natural state reserve BOSCO NEGRI
Objective:	eradication
Organism name:	Balkan Frog (*Pelophylax kurtmuelleri*)
Assessor name(s):	Adriana Bellati
Date / version:	28-08-23

**Title**	**Response**	**Confidence**	**Justification**
1. Define the scenario	*The species is present in the area at low abundance and it has not been recorded in the surrounding area, therefore the presence of a single population can be postulated. The risk assessment area is almost a closed environment, with connection to freshwater inlet or outlet (Ticino river)*		
2. Define the eradication strategy	Trapping/Removal		
3a. How effective is the strategy?	5 - V EFFECTIVE<br>4 – EFFECTIVE<br>3 – MODERATE	3 – HIGH<br>2 – MED<br>1 – LOW	Although no data exists for the target species, evidence of its applicability for the successful

	2 – INEFFECTIVE<br>1 - V INEFFECTIVE		eradication of other amphibians exists in the literature (Khars, 2006; Hegan, 2014; Kamoroff et al., 2019). The main constrain concerns its cryptic morphology compared to native taxa (particularly *P. esculentus*), and possible hybridization of the target species with native taxa occurring in the area (Holsbeek & Jooris, 2010). Therefore, molecular analysis is needed to support morphological identification.<br>The strategy could then be considered moderately effective, if applied timely, i.e. shortly after the detection of a few individuals in a very small area corresponding to a closed environment, prior to or at the very beginning of the reproductive season
3b. How practical is the strategy?	5 - V PRACTICAL<br>4 – PRACTICAL<br>3 – MODERATE<br>2 – IMPRACTICAL<br>1 – V IMPRACTICAL	3 – HIGH<br>2 – MED<br>1 – LOW	Frogs could be trapped at night by hand using flashlights and fishnets, or by fish traps/pots, and seines during the day. Sampling procedure is easy in the field, but specific equipment and professional staff is required for identification of morphological diagnostic characters and particularly for the collection of biological samples for subsequent genetic analysis
3c. How expensive is the strategy?	5 (<£50K)<br>4 (£50-200K)<br>3 (£200K-1M)<br>2 (1-10M)<br>1 (> £10M)	3 – HIGH<br>2 – MED<br>1 – LOW	Direct costs are mainly related to: recruitment of specialized staff (herpetologists); repeated sampling sessions (several days along the reproductive season); genetic analysis. It could be sustainable only if used for a closed and not too broad environment
3d. How much negative impact would the strategy have?	5 – MINIMAL<br>4 – MINOR<br>3 – MODERATE<br>2 – MAJOR<br>1 – MASSIVE	3 – HIGH<br>2 – MED<br>1 – LOW	The proposed strategy has low impacts on the aquatic environment and its biota, as non-target species can be immediately released in the wild after occasional trapping (e.g., by fishnets), while native species

			could be released in a few days after genetic screening
3e. How acceptable is the strategy?	5 - V ACCEPTABLE<br>4 – ACCEPTABLE<br>3 – MODERATE<br>2 – UNACCEPTABLE<br>1 - V UNACCEPTABLE	3 – HIGH<br>2 – MED<br>1 – LOW	Although the proposed strategy is the only one applicable to eradicate the species, frog capture can raise ethical problems as the species is well-liked by local people, therefore it should be important to inform the local stakeholders before proceeding.
4. What is the window of opportunity for implementing the strategy?	5 (10+ YRS)<br>4 (4-10 YRS)<br>3 (1 – 3 YRS)<br>2 (2 MTHS - 1 YR)<br>1 (< 2 MTHS)	3 – HIGH<br>2 – MED<br>1 – LOW	The species has a very high reproduction rate, laying numerous eggs, with a high hatching and development success. Available information concerning the survival of possible hybrids suggest high risk of genetic pollution of the native populations if the eradication strategy is not applied timely. Similarly, if the area is not sufficiently closed, new individuals could colonize the area every year, vanishing the effectiveness of the strategy
5. What is the likelihood of reinvasion?	5 – V UNLIKELY<br>4 – UNLIKELY<br>3 – MODERATE<br>2 – LIKELY<br>1 – V LIKELY	3 – HIGH<br>2 – MED<br>1 – LOW	The assessment area is basically a closed pond, but it is very close to the main river and it is surrounded by the secondary hydrographic system which is invaded by the species. As the species probably colonized the area via the Ticino river from their primary invasion range (south to the Po river), the likelihood of reinvasion of the wetland is quite high
6. Conclusion (overall feasibility of eradication)	5 – V HIGH<br>4 – HIGH<br>3 – MEDIUM<br>2 – LOW<br>1 – V LOW	3 – HIGH<br>2 – MED<br>1 – LOW	As the wetland is ideally in connection with the current northern edge of the invasion species range in the Po Plain (Bellati et al., 2023), and the species is some way cryptic in the wild, the feasibility of the eradication should be scored as "low", although if the strategy is applied timely and rigorously, it would have much more chances to be successful
